# Supplementary material for: Targets for the diagnosis of Acanthamoeba eye infections include four cyst wall proteins and the mannose-binding domain of the trophozoite mannose-binding protein
Source: mSphere. 2025 Mar 4;10(3):e00948-24. doi: 10.1128/msphere.00948-24 (PMC11934332; doi:10.1128/msphere.00948-24)
Supplement: Supplemental figures — Fig. S1 to S3. [file msphere.00948-24-s0002.docx]

Supplemental Figures for “Targets for the diagnosis of *Acanthamoeba* eye infections include four cyst wall proteins and the mannose-binding domain of the trophozoite mannose-binding protein.”

Bharath Kanakapura Sundararaj, Manish Goyal, and John Samuelson

Fig. S1. Sequences of Jonah-1, Luke-2, Leo-A, laccase-1, and ManBD used for making *E. coli* maltose-binding protein-fusions for immunizing rabbits.

Fig. S2. Alignment of CuRO-1 domains of laccases of *Acanthamoeba* and selected bacteria.

Fig. S3. Neighbor-joining tree of 18S RNAs of 11 *Acanthamoeba* isolates.

>Jonah-1 (ACA1_164810)

VGSSCPAEDFFGPAADLNVLVTGSDKLIYDLDMVNGDVEGRVAVNGGFRVKSFGTAQAYSCPDTANYASMFNLIVNGKMDYSNGQLFCGSSISLSSMNDPSLRQKDFGATNARVAAGKTIKDVTGFDFVATAGYLQGVSNYFSKYAPGTHSHFRVTEVSPADVNKLNIEGGDYVQVINIKGSGDINFSNFEIPTALNPSQVIYNVVGNNNIKISGFGLKGHLLALNSDVVLENGHVAGNVYVRSLVGQGSGQVNLAPCP

>Luke-2 (ACA1_377670)

QSCTASLNQTRQASWVDSEQFPRSLWTVEIRNTGQQAVTNVLLSIQGSINQIWEVVLVNDSLYRLPDWRLQVGGIPAGQSHVFGFIVNNNSTAAPVSLVSVQCGNVTVPSPSPSASASSVPSPSASASAAPSPSAGASPSSSPRPSPSGGCSLLASQVARSGAGGSWSDNSNRFQIYDITLNNNGASRLTQASLTIAIAADQAIAQFWNLERVDATNTFNIESFLLPAPGGSQSGLGYVLQTPLNSTSDGSSIGAVNFRC

>Leo-A (ACA1_074730)

CPGSDFFPDQRGCCPVVVNSVPFYADAQGCFPRIISGVAFYADAGGCYPSSAGVYRSAGAVCPADRVCEPECPDMAIVVAEQQGGARLCANGFFSDVNGCCPRVLVDNVPSYRDAQGCYPISIASVIFYADVGRCYPNSSGVYRPAGAVCPAGQQCVVSCDRNYATS

>laccase-1 (ACA1_068450)

VKQTCDGDHYDITMGVVKHQFHPSLPVVDIYGYGGTYPGGTFEAVVNRPVHVTWRNSLPEKHILPTEPLEMEGMHMENPPDSAGVVHLHGALIDPKDDGFSMDWITRGQKIQYHYTNEQLPLTMWYHDHVMTRTRVNVYAGLAGFYLLRDE

>ManBD of AcMBP-1 (AAT37864)

GTCNLSGAIKQPGLDCSSTSCSITSGTFPFPLPQGETYDSFYSWILGVIGTDGATVNAQYVDYTKADPNIYFTAGQTNCMVNLTFVYEVAFYRNSMGYFTFTRDSKPTSVGSVTLKPVFSETTVDCSRTSSQPLPGTSCLAPGSTISLGPFSSTQAVGFYLKQDSICSGTTTFYSVDALNKVTSRWKPIPAAHGRMIAVLRDPNTLRAYLGFEDSPDGSDSDYNDNVFSVTSNCEIDTSLLP

**Fig. S1. Sequences of Jonah-1, Luke-2, Leo-A, laccase-1, and ManBD used for making *E. coli* maltose-binding protein-fusions for immunizing rabbits.** Jonah-1 contains a single β-helical fold, while Luke-2 contains two β-jelly-roll folds separated by Ser-rich spacer. Leo-A contains two sets of four disulfide knots, while laccase-1 contains the first copper oxidase domain. The mannose-binding domain (ManBD) of the *Acanthamoeba* mannose-binding protein (AcMBP-1, which is absent in AmoebaDB but present in NR database of NCBI), contains an antiparallel β-sandwich.

Ac laccase-1 VKQTCDGDHYDITMGVVKHQFHPSLPVVDIYGYGGTYPGGTFEAVVNRPVHVTWRNSLPE 60

Ac laccase-2 YCTRRGVDKYVVTMSAIQHRLHPDLNLTQVYGYAGTYPGGTIEAVVDRPVAVTWRNHLPD 60

Ac laccase-3 VSRKCGVDRYEVTFNSFEQQLHPSLPPTTVYGYDGSYPGATFEAKVNRPVEVTWTNDLPL 60

Ac laccase-1 KHILPTEPLEMEGMHMENPPDSAGVVHLHGALIDPKDDGFSMDWITRGQKIQYHYTNEQL 120

Ac laccase-2 THFLPTEPLGHLG--SEPVPDSAAVPHLHGAHVPPDSDGDPMVWITRGQDADFYYPNGQL 118

Ac laccase-3 HHILPTVPLEG----SETPPESAGVVHLHGGHTPADSDGNPLDFIVKGQQQQFHYPNRQL 116

Ac laccase-1 PLTMWYHDHVMTRTRVNVYAGLAGFYLLRDE 151

Ac laccase-2 ATTMWYHDHIMGRTRVNVYAGLVGFYLLRDE 149

Ac laccase-3 ATTLWYHDHIWTRNRANVVAGLAGFYLLRDK 147

*Clostridium* GTTMWYHDHGMGETRLNVYAGLAGFYILRDS 153

*Bacillus* QAILWYHDHAMALTRLNVYAGLVGAYIIHD- 158

*Bifiguratus* PATMWYHDHALGITRLNVYAGLVGFYLLR-- 150

*Daucus* PGNLWYHDHAMGLTRVNLLAGLIGSYIIR-- 150

**Fig. S2. Alignment of CuRO-1 domains of laccases of *Acanthamoeba* and selected bacteria**. For clarity, only the C-terminal sequences of the CuRO-1 domain of bacteria are marked, while yellow marks amino acid identities with CuRO-1 domain of *Acanthamoeba* laccase-1. Ac laccase-2 (ACA1_006180) had minor errors in protein prediction in AmoebaDB, which were corrected using an unpublished transcriptome and proteome made in collaboration with investigators at the Broad Institute. Ac laccase-3 (ACA1_008840) was severely truncated in AmoebaDB and so lacked the CuRO-1 domain, which was identified in the unpublished transcriptome and proteome. Other CuRO-1 sequences came from *Clostridium sp.* (SFU68967), *Bacillus cenocepacia* (4AK0), *Bifiguratus adelaidae* (OZJ01666), and *Daucus carota* (XP_017216893).

**
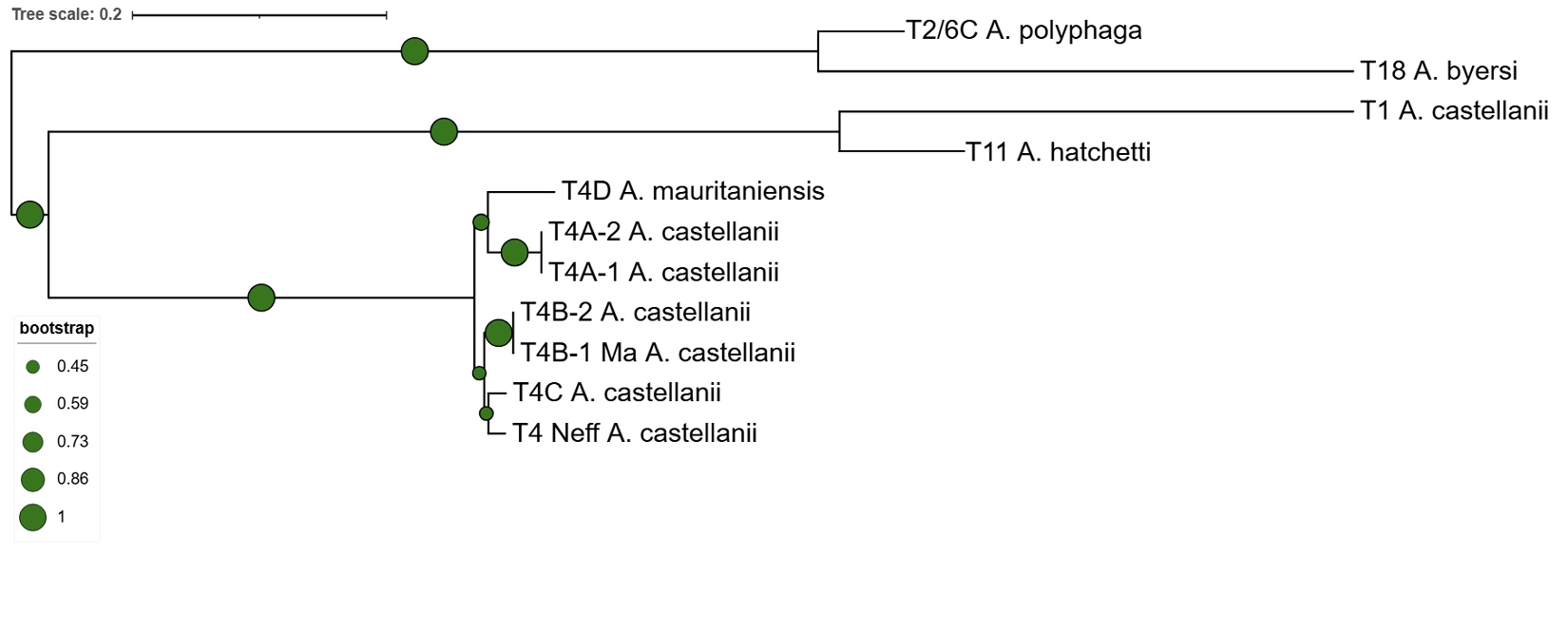
Fig. S3. Neighbor-joining tree of 18S RNAs of 11 *Acanthamoeba* isolates.** This tree shows that 18S RNAs of two pairs of isolates of *A. castellanii* (T4A-1/2 and T4B-1/2) are each identical to each other, and all T4 isolates are similar. In contrast, 18S RNAs of T1, T11, T18, and T2/6C are all different from each other. See Excel file S1 for further information about Acanthamoeba genotypes.
